# Impacts of Climate Change on the Spatial Distribution and Habitat Suitability of *Nitraria tangutorum*

**DOI:** 10.3390/plants14101446

**Published:** 2025-05-12

**Authors:** Lianxing Li, Zhiqing Jia, Lingxianzi He, Dong Han, Qiankun Yang, Jialuo Li, Pingyi Zhou

**Affiliations:** 1 Research Institute of Forestry, Chinese Academy of Forestry, Beijing 100091, China; li.lianxing@outlook.com (L.L.); handong7@outlook.com (D.H.); qkyangds@caf.ac.cn (Q.Y.); lijialuo@caf.ac.cn (J.L.); emma561561@163.com (P.Z.); 2 Institute of Ecological Conservation and Restoration, Chinese Academy of Forestry, Beijing 100091, China

**Keywords:** *Nitraria tangutorum*, MaxEnt, species distribution, climate change, potential distribution prediction

## Abstract

*Nitraria tangutorum* (Zygophyllaceae) is an ecologically and economically valuable shrub, locally dominant in the arid and semi-arid deserts of northwest China owing to its exceptional drought resistance and salt tolerance. In this study, environmental variable importance was evaluated within the MaxEnt model using percent-contribution metrics, based on 154 distribution records of *N. tangutorum* and 14 bioclimatic and soil-related environmental variables. We identified the five key variables of *N. tangutorum* distribution as follows: Precipitation of the Wettest Quarter (Bio16), Topsoil Sodicity (T_esp), Topsoil Electroconductibility (T_ece), Topsoil Car-bonate or lime content (T_CACO_3_), and Precipitation of the Driest Month (Bio14). The constructed MaxEnt model was then used to project the potential distribution areas of *N. tangutorum* under the four Shared Socioeconomic Pathways (SSP1-2.6, SSP2-4.5, SSP3-7.0, SSP5-8.5) for both current climate conditions and future climate conditions (2050s and 2090s). The results indicate that, under present-day conditions, high-suitability areas occur primarily in Xinjiang, Gansu, Qinghai, Inner Mongolia, and Ningxia; in future climate scenarios, the suitable habitat for *N. tangutorum* is anticipated to shrink by the 2050s but is expected to gradually recover by the 2090s. As time progresses, the suitable habitat areas will generally expand towards higher latitude regions. These findings demonstrate *N. tangutorum*’s strong adaptive potential to climate change and provide a scientific basis for its targeted introduction, cultivation, and long-term management in desert restoration and ecological rehabilitation projects.

## 1. Introduction

As a pivotal determinant of biodiversity, climate governs the growth, development, and geographical distribution of organisms [1]. Anthropogenic greenhouse gas emissions generated by societal progress have increased the frequency of extreme weather events, thereby imposing significant societal burdens. Simultaneously, the continually changing hydrothermal conditions have also disrupted the distribution of species [2,3]. Yet, plants can acclimate to climate change through phenotypic plasticity, such as modifications in phenological stages [4]. Under projected climate scenarios, plant populations unable to persist in their native environments typically migrate toward higher latitudes and altitudes, though a subset of species may exhibit reverse shifts to lower elevations and latitudes [5,6]. Species possess a limited capacity to adapt to climate change; however, if they cannot adapt or migrate to suitable habitats, some or even entire populations are at risk of extinction [7]. Therefore, investigating species distribution patterns enhances our understanding of how climate adaptation influences geographical ranges, providing a scientific foundation for biodiversity conservation and sustainable species management [2].

Species distribution models (SDMs), which utilize existing distribution data and environmental variables, are commonly employed to project species’ geographical patterns under climate change scenarios [8]. Among these frameworks, the Maxent model is widely recognized as a leading tool, leveraging machine learning principles and maximum entropy theory to estimate species’ potential geographic ranges [9]. Notably, even under conditions of poorly defined environmental relationships or sparse distribution data, Maxent enhances predictive accuracy, rendering it well-suited for species distribution modeling research [10]. While Maxent demonstrates robust performance in species distribution prediction, studies highlight that its default parameter settings can substantially influence outcomes. Thus, implementing the ENMeval package for parameter optimization effectively improves prediction precision [11].

*Nitraria tangutorum* is a hardy, drought-resistant shrub in the genus *Nitraria* of the Zygophyllaceae family, native and endemic to China [12]. This species exhibits exceptional resilience to harsh environmental conditions and robust regenerative capabilities. It has a well-developed root system and numerous branches, making it highly effective at stabilizing shifting sands and reducing wind speed. This species is a dominant and key constructive species in the arid desert regions of northwestern China, serving as an excellent plant for windbreak and sand-fixation purposes [13,14]. In addition to its exceptional ecological value, *N. tangutorum* also possesses significant economic and medicinal value [15]. Its fruit is referred to as the “desert cherry” due to its cherry-like color, shape, and flavor, and is rich in vitamins, amino acids, sugars, and trace elements [16]. Medicinally, it is used to strengthen the spleen and stomach, alleviate digestive disorders, and modulate blood pressure and glucose levels [12,17]. The leaves of *N. tangutorum* are high in protein and low in fiber, making them excellent forage with superior feed quality, suitable for advancing livestock farming [18]. Moreover, *N. tangutorum* acts as a host plant for the valuable medicinal species *Cynomorium songaricum*, further augmenting its economic significance [19]. However, projected climate changes and socio-economic developments may alter its distribution patterns. As a desert plant with pivotal ecological restoration value and promising economic potential, investigating climate change impacts on *N. tangutorum*’s distribution is critical for understanding resilient plant species’ dynamic responses to environmental shifts, underscoring the importance of this research.

Based on this, this study uses optimized parameters to construct the Maxent model, which investigates the main environmental factors influencing the distribution of *N. tangutorum*. It forecasts the geographical distribution of *N. tangutorum* under both current and various future climate scenarios, aiming to analyze the dynamic shifts in its suitable habitat. This research aims to develop effective conservation strategies that provide insight into the ecological adaptability of *N. tangutorum* and a basis for the rational use and protection of its germplasm resources.

## 2. Materials and Methods

### 2.1. N. tangutorum Distribution Point Collection

**Figure 1:**
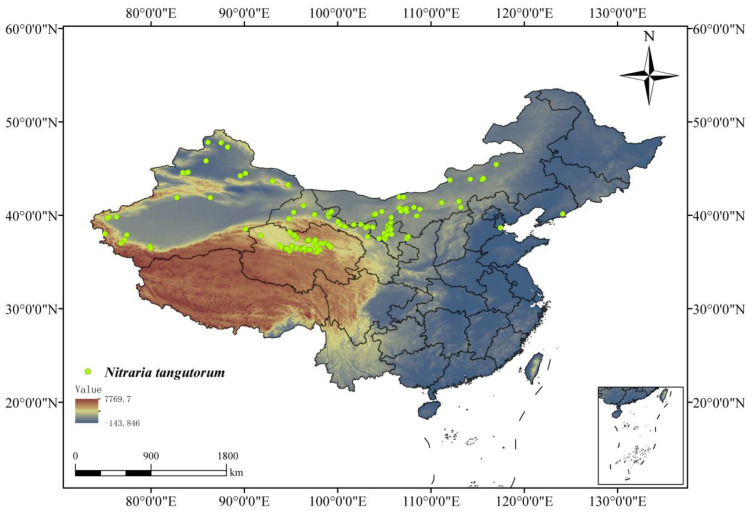
Location of *Nitraria tangutorum* sample points.

Geographical distribution data for *N. tangutorum* were obtained from the Global Biodiversity Information Facility (GBIF), the Chinese Virtual Herbarium (CVH), and the National Specimen Information Infrastructure (NSII), supplemented by field surveys and validated against the Flora of China and the relevant literature. To ensure data reliability, duplicate records and ambiguous data points were excluded. To reduce sampling bias and improve prediction accuracy, only one unique distribution point per 2.5′ × 2.5′ grid cell was retained [20]. The final dataset consisted of 154 species distribution points used in the model calculations (Figure 1).

### 2.2. Environmental Variable Screening and Processing

This study utilized 19 bioclimatic factors (bio1–bio19), 16 soil factors, and 3 topographic factors to develop species distribution models. Meteorological data were sourced from the World Climate Database (https://www.worldclim.org), covering three periods: the present (1970–2000), the future (2041–2060, 2050s), and the future (2081–2100, 2090s), with a spatial resolution of 2.5′. Projections of future climate conditions were derived from the BCC-CSM2-MR model, a component of the Coupled Model Intercomparison Project Phase 6 (CMIP6). The analysis incorporated four Shared Socio-economic Pathways (SSPs): SSP1-2.6, SSP2-4.5, SSP3-7.0, and SSP5-8.5 [21]. The BCC-CSM2-MR model has significantly improved the simulation of precipitation distribution in China and processes such as radiation budget, sea surface temperature, sea ice, stratospheric quasi-biennial oscillation (QBO), Atlantic meridional overturning circulation (AMOC), and Madden-Julian oscillation (MJO), enabling effective and accurate simulations of extreme temperature indices and trends in the region [22,23]. Soil factor data used in this study were obtained from the World Soil Database (https://www.fao.org/) at a spatial resolution of 2.5′. Environmental layers were converted to ASCII format using the SDMtoolbox plugin in ArcGIS 10.8 software [24].

To avoid multicollinearity among selected bioclimatic variables, which could cause model overfitting and result in unreliable species distribution predictions, the filtered *N. tangutorum* distribution data, along with standardized environmental variables in uniform format and resolution, were imported into the MaxEnt model. The model was run ten times to establish an initial model, and environmental variables with a contribution rate below 1% were excluded based on the averaged results. Subsequently, the correlation tool in ENMtools v1.4.4 software was used to analyze the relationships among the remaining environmental factors. When two environmental factors showed high correlation (|r| > 0.8), only the variable with the highest contribution rate was retained for model prediction [25]. Following these steps, fourteen environmental factors were finally screened.

### 2.3. Establishment, Optimization, and Evaluation of the MaxEnt Model

The performance of the MaxEnt model is highly contingent on parameter configuration, as different parameters significantly influence prediction outcomes. Feature Combination (FC) and Regularization Multiplier (RM) are critical parameters in this context. Optimizing these parameters aids in minimizing overfitting and substantially enhances the model’s predictive performance [26]. Parameter optimization via the ENMeval package in R v4.3.0 software reduces model complexity and overfitting, enabling more accurate predictions of species’ potential distribution areas [27]. The ENMeval v2.0.4 package was used to tune RM and FC parameters for the MaxEnt model [28]. The RM was set at eight levels ranging from 0.5 to 4.0, with 0.5 increments between levels. Six feature combinations were employed (H, L, LQ, LQH, LQHP, LQHPT), yielding 48 total combinations. Model accuracy and goodness-of-fit were evaluated using the Akaike Information Criterion (Delta.AICc). Model fit was assessed via the average difference between training-set AUC and test-set AUC (Avg.diff.AUC) and the mean value of the 10% training omission rate (Mean.OR10). The model with the lowest Delta.AICc value was selected as optimal [29].

In this study, MaxEnt 3.4.1 was used to project the potential suitable habitats of *N. tangutorum* across different time periods. Selected environmental variables and species distribution data were input into the MaxEnt model, configured with the optimal parameter combination identified earlier. For model training, 75% of data was randomly selected as the training set, while 25% was reserved as the test set to validate predictive accuracy. The model was set to run for a maximum of 500 iterations with 10,000 background points. It was executed ten times using the Bootstrap method, with the average results recorded. To assess model performance, a Receiver Operating Characteristic (ROC) curve was generated, providing a measure of the model’s fit [30].

The Area Under the ROC Curve (AUC) value is unaffected by the threshold and serves as a quantitative measure for diagnosing the performance and strength of the model. Ranging from 0 to 1, higher AUC values indicate greater predictive accuracy [31]. Specifically, models with AUC ≤ 0.7 are considered unreliable; those with 0.7 < AUC ≤ 0.8 exhibit moderate predictive capability; 0.8 < AUC ≤ 0.9 signifies high accuracy; and AUC > 0.9 denotes exceptional predictive precision, enabling detailed characterization of species’ potential distributions [32].

### 2.4. Division of Potential Suitable Habitats for N. tangutorum

The output of the MaxEnt model was converted to a raster file in ArcGIS and then reclassified. Using the Natural Breaks classification method, suitability was divided into four categories: no suitability, low suitability, medium suitability, and high suitability areas. The proportion of each suitability zone was calculated using the raster layer attribute table in ArcGIS software, and the area of each category across different time periods was computed [33].

### 2.5. Centroid Migration of the Suitable Area

The dynamic shifts in suitability area centroids were analyzed using the SDMtoolbox v2.5 within ArcGIS v10.8. The “Centroid Changes (Lines)” tool was employed to compute a single centroid for each period’s sub-regions of *N. tangutorum*. Temporal variations in centroid locations reveal the migration trends and spatial shifts of the species’ suitable habitats [20].

## 3. Results

### 3.1. Model Optimization Results and Evaluation

To minimize the risk of model overfitting and ensure accurate predictions, this study utilized the ENMeval package to conduct cross-validation on the RM and FC (Table 1). A MaxEnt model was developed using 14 environmental variables and 154 *N. tangutorum* distribution points to simulate potential distribution areas. Setting default parameters (RM = 1, FC = LQHP) yielded a Delta.AICc value of 967.58, whereas optimized parameters reduced Delta.AICc to 0, with lower Mean.OR10 and Avg.diff.AUC values compared to the default model. These results indicate improved model performance, leading to the selection of the optimized parameter combination (RM = 3, FC = LQHPT) for final modeling based on the Akaike Information Criterion.

**Figure 2:**
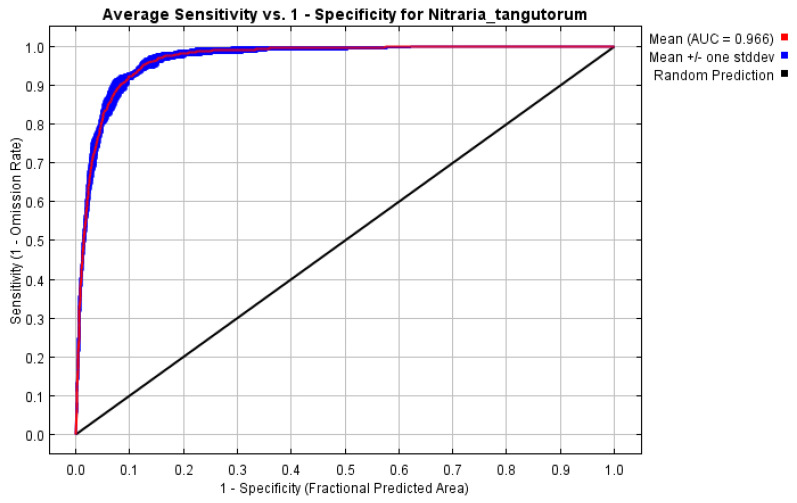
The ROC curve predicted by the MaxEnt model.

The optimal parameters were used in the MaxEnt model to perform ten simulations of the potential suitable habitats of *N. tangutorum*. The training AUC and test AUC of the model were calculated based on the ROC curve (Figure 2). The test AUC values ranged from 0.963 to 0.9687, with an average of 0.9662 ± 0.0018. Training AUC values spanned 0.895 to 0.9715, averaging 0.951 ± 0.0223. Collectively, these results demonstrate the model’s high predictive accuracy and robust reliability, confirming its suitability for characterizing species distribution dynamics.

### 3.2. Key Environmental Variables Affecting the Distribution of N. tangutorum

The influence of environmental variables on *N. tangutorum* suitable habitat distribution was evaluated through a comprehensive analysis of variable contribution rates and Jackknife test results. Contribution rate rankings identified the top five variables as Bio16, T_esp, T_ece, T_CACO_3_, and Bio14, collectively accounting for 71.4% of total contribution (Table 2). Among topographic factors, only DEM significantly impacted natural *N. tangutorum* distributions. Permutation importance—calculated by randomly permuting variable values in background training data—measures dependency strength, where higher values indicate greater influence. The top five variables by permutation importance contributed a cumulative 72.3% to model predictions. Overall, these results highlight precipitation and soil factors as primary drivers of *N. tangutorum* distribution patterns.

**Figure 3:**
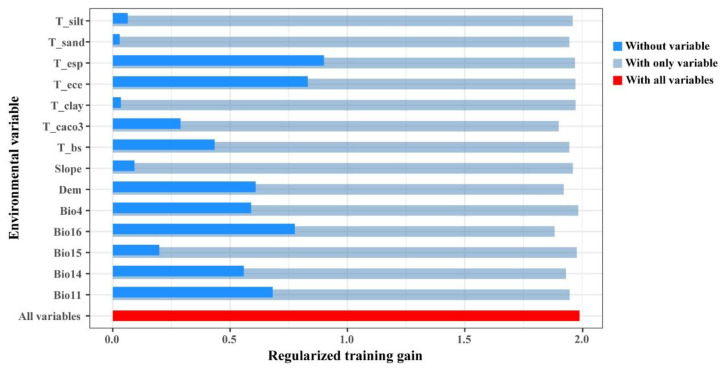
The results of the Jackknife test for the environmental factors.

The Jackknife test indicated that the environmental variables T_esp, T_ece, Bio16, Bio11, and DEM had the most significant impact on regularized training gain when used individually. This suggests that these environmental variables contain unique information not captured by other variables and play a crucial role in determining the distribution of suitable habitats for *N. tangutorum* (Figure 3).

**Figure 4:**
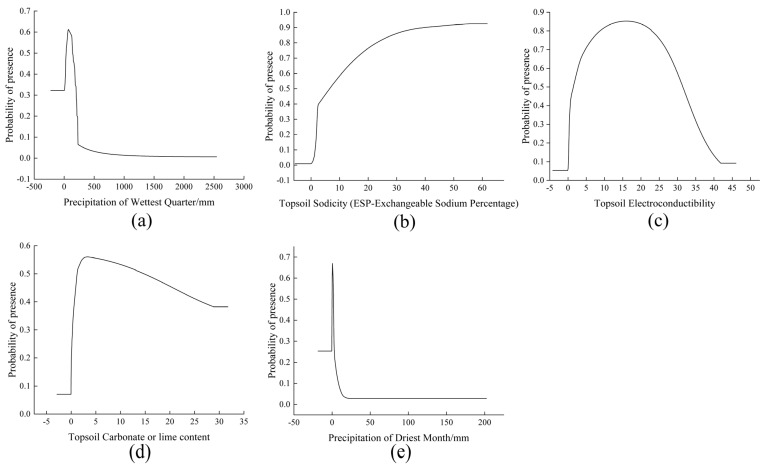
Response curves of main bioclimatic factors. (**a**) The reaction curve of Precipitation of Wettest Quarter(Bio16) and the probability of presence; (**b**) The reaction curve of Topsoil Sodicity (T_esp) and the probability of presence; (**c**) The reaction curve of Topsoil Electroconductibility (T_ece) and the probability of presence; (**d**) The reaction curve of Topsoil Carbonate or lime content (T_CACO_3_) and the probability of presence; (**e**) The reaction curve of Precipitation of Driest Month (Bio14) and the probability of presence.

Plotting response curves for environmental variables clarifies the relationship between each factor and species distribution probability, facilitating the determination of *N. tangutorum*’s potential habitats. Among the variables included in the model, Bio16, T_esp, T_ece, T_CACO_3_, and Bio14 emerged as the most influential determinants of distribution, with their response curves shown in Figure 4. For all variables except T_esp, the probability of *N. tangutorum* occurrence increases with rising variable values, reaching a peak before declining as conditions exceed optimal thresholds. Within a specified interval, occurrence probability exhibits a positive correlation with T_esp, characterized by an initially steep increase followed by a gradual slowdown in growth rate. These results reinforce that precipitation (Bio16, Bio14) and soil properties (T_esp, T_ece, T_CACO_3_) are primary environmental drivers shaping *N. tangutorum*’s distribution patterns. Furthermore, using probability thresholds of 0.3 and 0.5 for the suitable habitat of *N. tangutorum* as criteria, suboptimal and optimal values for the dominant environmental variables were derived (Table 3). When environmental variable values fall within predefined optimal thresholds, the area is considered to satisfy the optimal growth conditions for *N. tangutorum* germplasm resources. The optimal threshold values (*p* > 0.5) for each environmental variable are as follows: Bio16 (34.17–139.32 mm), T_esp (6.58–61.74%), T_ece (1.49–31.43 dS·m^−1^), T_CACO_3_ (1.20–14.71%) and Bio14 (0.15–1.91 mm).

### 3.3. The Current Suitable Habitat Distribution of N. tangutorum

**Figure 5:**
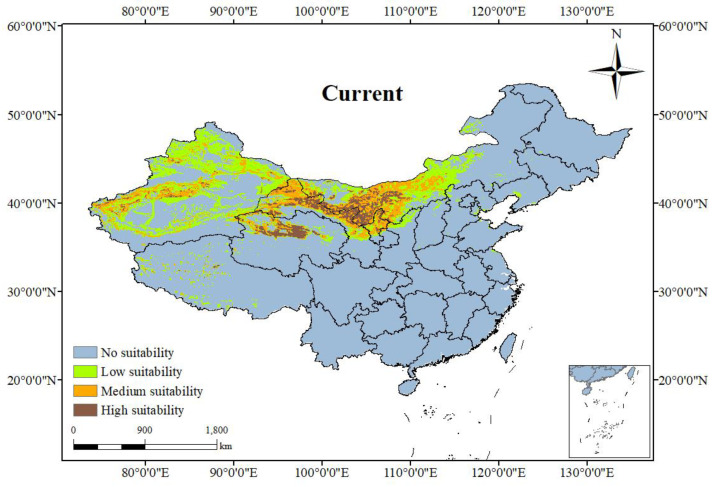
The suitable habitat of *N. tangutorum* under current climate conditions. The blue grey areas represent no suitability habitats, green areas represent low suitability habitats, orange areas represent medium suitability habitats, and brown areas represent high suitability habitats.

Following the optimization and implementation of the MaxEnt model, the potential suitable habitats for *N. tangutorum* under current climate conditions were predicted (Figure 5). The figure indicates that high-suitability areas for *N. tangutorum* are predominantly distributed in northwestern China, encompassing central and western Gansu, central and western Inner Mongolia, the Qaidam Basin in Qinghai, central and western Xinjiang, and central-northern Ningxia. The total area is 24.21 × 10^4^ km^2^, accounting for 12.72% of the total suitable area. Medium-suitability areas are primarily located in central Inner Mongolia, south-central Gansu, northern and southern Xinjiang, and parts of Tibet, with a total area of 57.73 × 10^4^ km^2^, comprising 30.34% of the total suitable area. Low-suitability areas are mainly found in Inner Mongolia, Xinjiang, and northern Shanxi, with scattered regions in Tibet, totaling 108.35 × 10^4^ km^2^, which accounts for 56.94% of the total suitable area. The model projections show that the predicted suitable habitats for *N. tangutorum* are concentrated in the arid and semi-arid zones north of the 400 mm annual precipitation line. The predicted potential distribution area is larger than the actual occurrence data of *N. tangutorum*, suggesting that under current climate conditions, these regions are suitable for the survival of *N. tangutorum*. If introduced and cultivated in these areas, the survival probability of *N. tangutorum* would be relatively high.

Upon comparing the actual distribution points of *N. tangutorum* with the predictions of potential distribution under current climate conditions, it becomes evident that over 80% of the effective distribution points fall within the medium and high suitability areas. This observation validates the relative accuracy of the model predictions.

### 3.4. The Future Suitable Habitat Distribution of N. tangutorum

**Figure 6:**
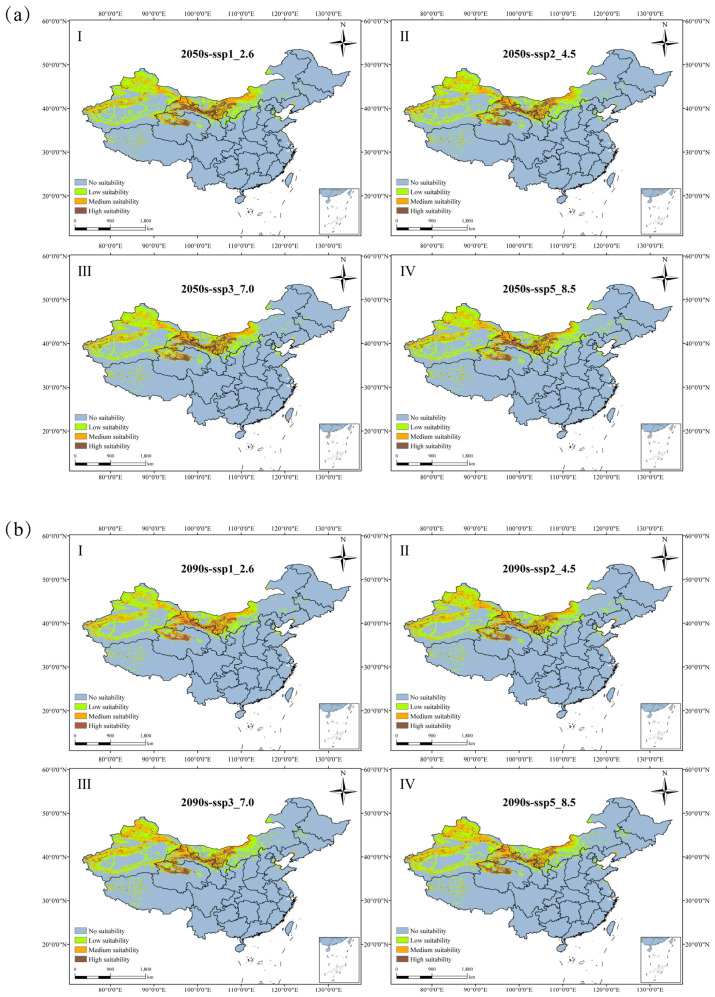
Projected habitats of *N. tangutorum* under four SSP scenarios (SSP1-2.6, SSP2-4.5, SSP3-7.0, SSP5-8.5) for the (**a**) 2050s and (**b**) 2090s. Subpanels I–IV in each panel (**a**,**b**) correspond to the four scenarios listed sequentially.

This investigation employs the MaxEnt modeling framework to forecast the prospective geographical distribution of *N. tangutorum* across four distinct future climatic scenarios, namely SSP216, SSP245, SSP370, and SSP585. The spatial delineation of potential habitats that are considered suitable is depicted in Figure 6, whereas the variations in the extent of habitat areas for each category are detailed in Table 4.

Comparing predicted habitat distributions of *N. tangutorum* under different future climate scenarios reveals divergent responses, with distinct trends in suitable area shifts. Under 2090s SSP370 and SSP585 scenarios, suitable habitat area is projected to expand relative to the current period, with expansion rates of 1.18% and 2.50%, respectively. In contrast, other future scenarios predict varying degrees of contraction. Across 2050s carbon emission scenarios, total suitable habitat area exhibits a declining trend compared to the present: projected reductions of 49,409 km^2^, 40,260 km^2^, 54,740 km^2^, and 16,145 km^2^ occur under respective scenarios. Relative to the 2050s, the 2090s see a 65,226 km^2^ reduction under SSP126, while SSP245, SSP370, and SSP585 scenarios drive increases of 25,834 km^2^, 77,171 km^2^, and 63,663 km^2^, respectively.

Under various emission scenarios for the 2050s and 2090s, the high suitability habitat area generally increases, except for a reduction under the 2090s-SSP126 scenario, which is characterized by a decrease of 14,513 km^2^ relative to the current period. The largest increase occurs under the 2050s-SSP245 scenario, with an expansion of 13.11%. The area of low suitability habitats in future periods is generally lower than the present period. However, the area changes in the 2050s under various scenarios are smaller compared to the 2090s. The most significant reduction occurs under the 2090s-SSP585, with a decrease of 66,614 km^2^.

There are different patterns of changes in the size of the medium suitability area under the future scenarios. In the 2050s, the area first decreases, reaching its lowest point under the 2050s-SSP370 at 53.87 × 104 km^2^. Subsequently, during the 2090s, as the greenhouse effect intensifies, the area of medium suitability area gradually increases, reaching its highest value under the 2090s-SSP585 at 65.72 × 104 km^2^.

**Figure 7:**
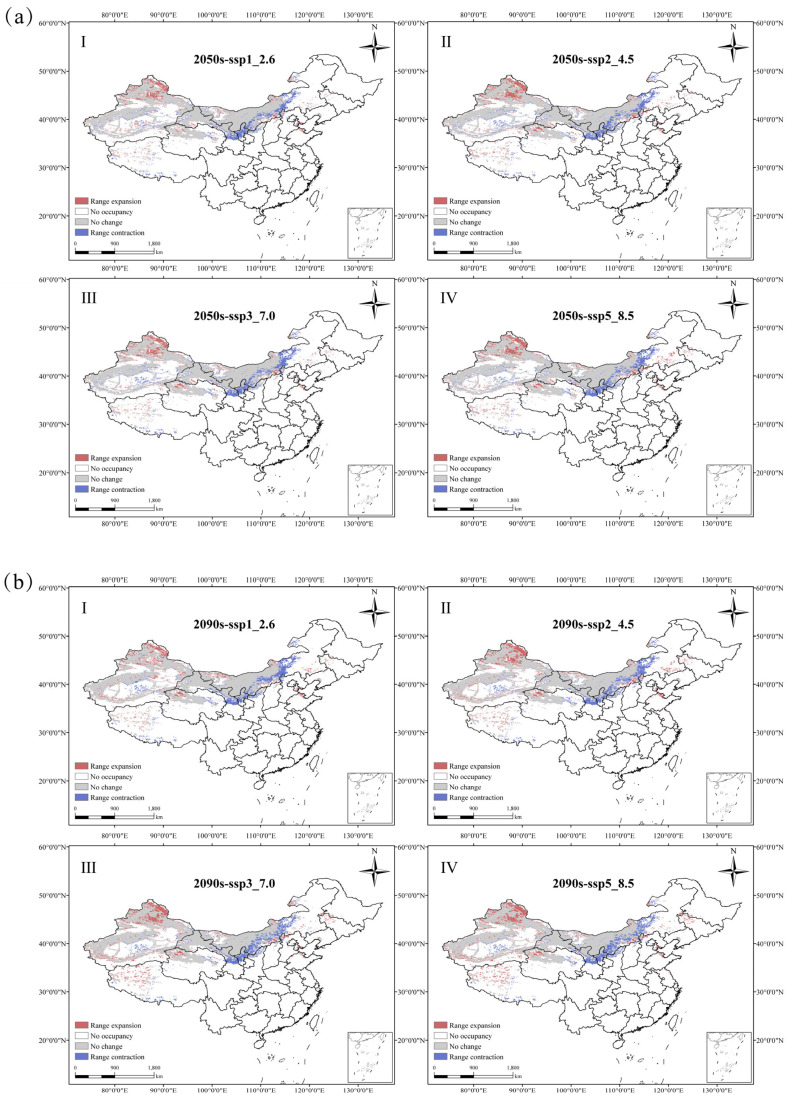
Spatial shifts in the geographic distribution of *N. tangutorum* under four SSP scenarios for the 2050s and 2070s. (**a**) 2050s; (**b**) 2070s. In each panel, I–IV denote SSP1-2.6, SSP2-4.5, SSP3-7.0 and SSP5-8.5, respectively; The red areas represent range expansion, white areas represent no occupancy, gray areas represent no change, and blue areas represent range contraction.

Analysis of the spatial pattern changes in *N. tangutorum* habitats under different future climate scenarios (Figure 7, Table 5) indicate that a majority of the current suitable habitats are maintained. Habitat retention rates remain within a narrow range of 79.41% to 80.85%, indicating remarkable stability amid climate fluctuations. These stable areas are predominantly concentrated in the Xinjiang, Inner Mongolia, Ningxia, Gansu, and Qinghai provinces. In the 2050s, retention rates of suitable habitats fluctuate between 79.42% and 80.85%, exhibiting a trend of initial decline followed by a slight increase. With rising emission levels, the area of contraction for suitable habitats consistently exceeds the area of expansion. The lost suitable habitats are primarily distributed in the southern part of Inner Mongolia, the central region of Ningxia, and the central areas of Gansu.

In the 2090s, under the SSP370 and SSP585 scenarios, the contraction rate of suitable habitats is greater than the expansion rate. In other periods, the contraction rate is consistently less than the expansion rate. During this period, the main areas of expansion are concentrated in northern Xinjiang and northern Qinghai. It is noteworthy that in the future, more suitable habitats for *N. tangutorum* are expected to emerge along the western coastal region of the Bohai Sea.

### 3.5. Centroid of Suitable Area of N. tangutorum Under Present and Different Future Climate Scenario

**Figure 8:**
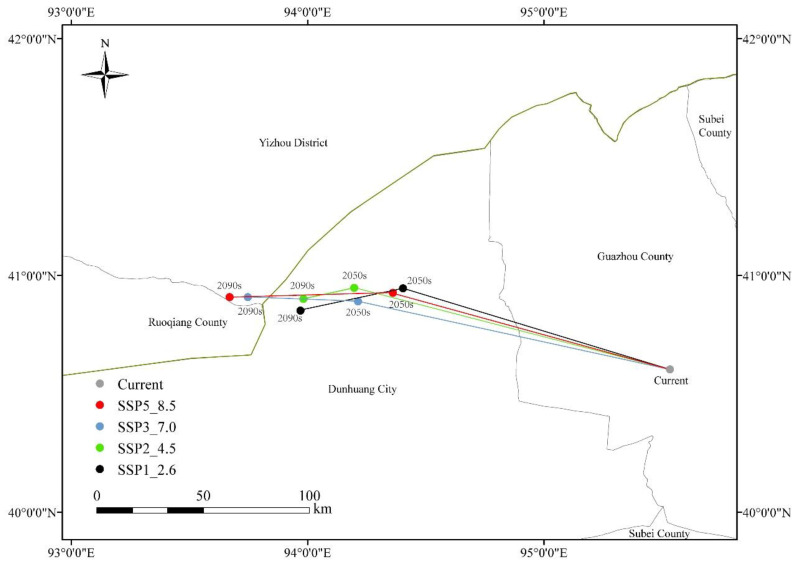
The migration of *N. tangutorum* centroid in different climatic scenarios.

As illustrated in Figure 8, ArcGIS software was employed to calculate the centroid of *N. tangutorum*’s suitable habitat under current and future climate scenarios, enabling analysis of centroid shift direction and distance. Overall, the centroid of the suitable habitat for *N. tangutorum* tends to shift westward under future climate scenarios as carbon emission concentrations increase, although the extent of this movement varies. The results indicate that under the current climate conditions, the centroid of *N. tangutorum* is located in Guazhou County, Jiuquan City, at coordinates 95.5324° E, 40.6036° N. Under the SSP126 scenario, by 2050, the centroid of *N. tangutorum* migrates 102.5 km northwest to Dunhuang City (94.4032° E, 40.946° N). By the 2090s, it continues to shift southwest by 38.02 km to 93.9691° E, 40.8512° N. The migration trend of the centroid under the SSP245 scenario is similar to that under SSP126, remaining within the Dunhuang City area. However, the migration distances differ. By the 2050s, the centroid shifts to 94.1965° E, 40.9479° N. By the 2090s, it further migrates to 93.9817° E, 40.9009° N, with a distance of 18.8 km between the two points. Under the SSP380 and SSP585 scenarios, as time progresses, the centroid initially migrates northwest and then shifts westward by the 2090s. Both centroids are located in Yizhou District, Hami City, Xinjiang, at coordinates 93.74663° E, 40.90884° N and 93.66997° E, 40.90806° N, respectively. Among all scenarios, the most significant migration distance occurs under the 2090s-SSP585 scenario, where the centroid shifts 160.67 km compared to its position in the current period.

## 4. Discussion

### 4.1. Accuracy Analysis of the Optimized Model

In this study, we used the optimised MaxEnt model combined with ArcGIS software, incorporating 154 distribution records of *N. tangutorum* and 14 environmental variables, to simulate the potential distribution areas of the species in China in the current and future periods. Evaluating species distribution with default parameters can result in overfitting and increased complexity, thereby reducing the accuracy of prediction results. This often manifests as significant fluctuations in the response curves of environmental factors [34]. Optimizing the MaxEnt model with the ENMeval package solves these issues by determining the optimal parameter configuration, effectively matching species distribution data with environmental variables for more reliable predictions. The resulting environmental variable response curves align with Shelford’s law of tolerance, effectively preventing overfitting [35]. Optimal MaxEnt model parameters were identified based on species characteristics and data structure. Generally, when the Regularization Multiplier (RM) ranges from 2 to 4, the model yields optimal performance, which aligns with our study’s optimal RM value of 3 [31,36]. ROC curve analysis revealed that all period-specific predictions for *N. tangutorum* exhibited AUC values exceeding 0.9, underscoring the model’s excellent predictive capability and high reliability. The potential suitable habitats of *N. tangutorum* under present climate conditions are mainly distributed in Gansu, Xinjiang, west-central Inner Mongolia, Qinghai, and Ningxia. The predicted results closely align with field survey data, further validating the reliability of the model’s predictions.

### 4.2. Effects of Environmental Variables on N. tangutorum Distribution

Based on the MaxEnt model’s contribution percentage, permutation importance, and Jackknife test results, the key environmental variables influencing the distribution of *N. tangutorum* include Precipitation of Wettest Quarter (Bio16), Topsoil Sodicity (T_esp), Topsoil Electroconductibility (T_ece), Topsoil Carbonate or Lime Content (T_CACO_3_), and Precipitation of Driest Month (Bio14). In the model construction, the total contribution rate of precipitation-related factors is 41.3%, while soil-related factors account for 40.8%. These findings underscore the significant role of both precipitation and soil characteristics as key limiting factors influencing the present distribution of *N. tangutorum*. This is consistent with the findings for other drought-resistant plants, such as *Tamarix taklamakanensis* and *Reaumuria kaschgarica*, where precipitation is also the primary climate-related limiting factor [37,38]. It was shown that precipitation during the wettest season typically determines topsoil moisture, which influences rapid root system growth in plant seedlings and thereby affects the survival of *N. tangutorum* seedlings [39]. Precipitation significantly affected the growth of *N. tangutorum* populations in Minqin County, a high-suitability habitat area. Annual precipitation of approximately 110 mm in the peripheral region of Minqin Oasis was found to maintain their normal growth and development, which aligns with the results of this study [40].

As a highly xerophytic plant, *N. tangutorum* has a higher probability of survival when precipitation of driest month, demonstrating a strong adaptation to arid environments [41]. In the main distribution areas of *N. tangutorum* such as the Qaidam Basin, Tengger Desert, and Ulan Buh Desert, dry conditions are common in late winter to early spring. As plants shift from dormancy to active growth during this period, their cold resistance decreases. Excessive precipitation during this time can affect the growth and development of *N. tangutorum*. As a result, the precipitation of driest month (Bio14) significantly contributes to the delineation of its suitable habitats. This highlights *N. tangutorum*’s unique adaptation to arid environments and explains why it has become a dominant species in the arid regions of northwestern China [38].

Topsoil Sodicity (ESP—Exchangeable Sodium Percentage), an indicator of soil salinity, indicates the ratio of exchangeable sodium ions to total cations in the soil, reflecting soil sodium salinity levels [42]. *N. tangutorum* exhibits a higher probability of occurrence in areas with elevated T_esp values. In such environments, sodium ions in exchangeable sodium salts reduce soil water conductivity and infiltration rates while fostering conditions of relatively low salt content and slight alkalinity. This enhances the plant’s ability to withstand osmotic pressure and improves its tolerance to salinity and alkalinity, explaining its remarkable salt-alkali resistance [43,44]. High concentrations of Na^+^ ions can lead to soil dispersion, affecting soil structure and reducing soil aeration and water retention capacity. However, *N. tangutorum* may counter these adverse factors through specific root structures or metabolic pathways, thus gaining an adaptive advantage in such environments [45,46]. The topsoil electrical conductivity reflects the level of soil salinity. As a desert plant with extremely high salinity-alkalinity tolerance, *N. tangutorum* can occupy ecological niches in soils that are highly alkaline and saline [47]. Although this study identified the key environmental factors influencing the growth of *N. tangutorum* and their response curves, data limitations restricted the analysis to abiotic factors such as climate, soil, and topography, without incorporating biotic factors (e.g., interspecific interactions) and anthropogenic influences (e.g., land-use change). Despite these limitations, the optimized MaxEnt model can still reliably predict the species’ potential suitable habitat range in China. Future research could further integrate biotic interactions and anthropogenic variables to refine the distribution model, providing more precise scientific guidance for the conservation planning and resource utilization of *N. tangutorum*.

### 4.3. N. tangutorum Geographic Distribution and Spatial Pattern Change

Under current climate conditions, the potential suitable habitats for *N. tangutorum* in China are primarily distributed across the provinces of Xinjiang, Qinghai, Gansu, Inner Mongolia, and Ningxia. This observation is broadly consistent with the outcomes of modeling suitable habitats for six species within the *Nitraria* genus utilizing the MaxEnt model. However, our predicted suitable habitat range is smaller, likely because this study focuses solely on *N. tangutorum*, whereas Duan’s study includes six species of the *Nitraria* genus, resulting in a broader distribution range. Moreover, by incorporating soil and topographical factors, our study generated more comprehensive predictions, which likely explain the discrepancies in the results [48].

Under future global warming scenarios, the suitable habitat range for *N. tangutorum* is expected to change somewhat, but there won’t be large-scale migration. The study’s findings suggest that with rising carbon emission concentrations, the suitable habitat area generally decreases by the 2050s but rebounds in some scenarios by the 2090s. This indicates that the effects of climate change on suitable habitat is not simply a linear reduction, but rather a dynamic change, highlighting the complexity of climate change effects on species habitat suitability. In low emission scenarios, the study indicates a continuous decline in the suitable habitat area for *N. tangutorum* in both the 2050s and 2090s, with particularly significant reductions in areas of low and high suitability. This phenomenon aligns with the findings of the *Pinus bungeana* potential habitat study, possibly because climate warming under the low-emission scenario reduces environmental conditions suitable for *N. tangutorum* growth [49]. The SSP585 scenario would see a significant increase in the area of suitable habitat by the 1990s, particularly in areas of medium to high suitability. This increase is likely due to the carbon dioxide fertilization effect resulting from higher CO_2_ concentrations under high emission scenarios, which promotes plant growth. However, the positive fertilization effect could be offset by extreme temperatures and water resource shortages [50]. During this period, the total suitable habitat area for *N. tangutorum* increases significantly. This could be due to the fact that under high emission scenarios, some plants within the suitable habitat of *N. tangutorum* may degrade and disappear as they fail to adapt to the changing environment. Consequently, the community’s species composition shifts as *N. tangutorum* occupies the ecological niches previously held by these plants. This pattern mirrors observations made in the desert plant *Hippophae rhamnoides* [51].

With ongoing global warming, plants and animals are expected to gradually shift to higher altitudes or latitudes to adapt to changing climates [52]. However, long-distance dispersal is limited in many plants [53]. The salty-sweet edible fruits of *N. tangutorum* are considered an important food source for animals; thus, its dispersal relies on birds, mammals, and occasionally ants [54]. This zoochorous trait may mitigate the impact of climatic changes by facilitating seed dispersal to newly suitable habitats, thereby explaining the model’s prediction of range recovery by the 2090s despite mid-century habitat shrinkage. The expansion of suitable habitats typically occurs at the margins of currently suitable areas, which serve as critical and sensitive zones where species respond dynamically to climate change [55]. The results indicate that *N. tangutorum* primarily expands towards Altay and surrounding areas in northern Xinjiang. Areas of habitat expansion concentrate in the northern portion of existing suitable zones, whereas contractions are primarily observed in the southern regions of current suitable habitats. This aligns with the trend of suitable habitats migrating towards higher lati-tudes under global warming [56,57]. Across different time periods, the retention rate of *N. tangutorum* suitable habitats remains above 79%, suggesting strong resilience of the species’ core distribution areas to future climate change. This is likely attributed to the species’ robust stress tolerance.

### 4.4. Protection Strategies

Studying the effects of climate change on the geographic distribution of species is valuable in providing scientific data to support species conservation [58]. Currently, the development and utilization of wild *N. tangutorum* remain limited, as it primarily inhabits harsh environments in its main distribution areas, with few established artificial populations. Wild germplasm resources typically exhibit high genetic diversity and possess valuable stress-resistant genes [59]. Therefore, based on the findings of this study, extensive collection of *N. tangutorum* germplasm resources holds significant importance for its future artificial cultivation and industrial development.

Under future climate scenarios, suitable habitats for *N. tangutorum* in southern Inner Mongolia, central Ningxia, and central Gansu are projected to gradually disappear. Consequently, the collection and conservation of wild germplasm resources in these regions are urgently needed. Translocating populations from these areas to moderately suitable regions for assisted migration will help mitigate adverse climate impacts, facilitate population reproduction and expansion, and enhance the species’ overall resilience and adaptability [60]. Furthermore, as suitable habitats for *N. tangutorum* are predicted to expand northward toward higher latitude regions, we recommend establishing additional nature reserves in expansion zones, such as northern Xinjiang, to accommodate future migration.

Additionally, we propose implementing incentive policies for artificial cultivation of *N. tangutorum* in high-suitability areas, including the Qaidam Basin, Hexi Corridor, and Alxa region. Establishing cultivation bases and developing *N. tangutorum* based industries in these areas will promote ecological conservation while increasing income for local communities. Our research not only enhances the conservation of *N. tangutorum* germplasm resources but also contributes to improving cultivation quality and the efficiency of ecological restoration efforts. This enables the species to maximize its ecological benefits and economic potential effectively.

## 5. Conclusions

This study identifies key environmental drivers of *N. tangutorum* distribution and reveals its dynamic responses to climate change. Precipitation extremes (Bio14, Bio16) and soil characteristics (T_esp, T_ece, T_CACO_3_) emerge as critical factors limiting its distribution, reflecting adaptations to aridity, salinity, and alkaline substrates. Current high-suitability areas are concentrated in arid/semi-arid regions of northwest China, aligning with its known drought- and salt-tolerant traits. At present, the total suitable area for *N. tangutorum* in China is 190.30 × 10^4^ km^2^, with the high suitability area comprising 12.72% of the total suitable area. Under future climate scenarios, suitable habitats exhibit a temporal shift: a moderate contraction by the 2050s, followed by a gradual expansion by the 2090s, with a notable northward shift toward higher latitudes. Although this study only used the MaxEnt model to simulate the suitable habitat distribution of *N. tangutorum*, the research results can offer valuable insights for studies on desert plant distribution, biodiversity, and the formulation of conservation strategies. These insights deepen our understanding of *N. tangutorum*’s responses to combined bioclimatic and soil environmental changes, providing a scientific basis for prioritizing the conservation of its germplasm resources and optimizing cultivation strategies. Future studies will incorporate more factors into the habitat prediction of *N. tangutorum*, further providing a theoretical foundation for the protection of its germplasm resources and scientifically guided planting management.

## Figures and Tables

**Table 1:** Comparison of model results between optimized parameters and default parameters.

Type	RM	FC	Delta.AICc	Mean.Diff.AUC	Mean.OR10
default	1	LQHP	967.58	0.052	0.21
optimized	3	LQHPT	0	0.04	0.20

**Table 2:** Percentage contribution and permutation importance of environmental factors.

Variables	Description	Percent Contribution	Permutation Importance
Bio16	Precipitation of Wettest Quarter	32.4	36
T_esp	Topsoil Sodicity (ESP-Exchangeable Sodium Percentage)	13.7	1.4
T_ece	Topsoil Electroconductibility	9.8	1.5
T_CACO_3_	Topsoil Carbonate or lime content	9.2	10.2
Bio14	Precipitation of Driest Month	6.3	5.3
Dem	Elevation	6	8.8
Bio11	Mean Temperature of Coldest Quarter	5.2	9.5
Bio4	Temperature Seasonality	5.2	1.5
T_bs	Basic saturation	3.6	6
Bio15	Precipitation Seasonality	2.6	1.7
T_sand	Topsoil sand content	2	7.8
T_silt	Topsoil silt content	1.5	5.2
Slope	Slope variability	1.3	1.7
T_clay	Topsoil clay content	1	3.4

**Table 3:** Optimal and suboptimal growing condition ranges of key environmental variables for *N. tangutorum*.

Variable	Suboptimal Ranges	Optimal Ranges
Precipitation of Wettest Quarter (Bio16)	0.96–200.20 mm	34.17–139.32 mm
Topsoil Sodicity (ESP-Exchangeable Sodium Percentage) (T_esp)	2.13–61.74%	6.58–61.74%
Topsoil Electroconductibility (T_ece)	0.39–35.40 dS·m^−1^	1.49–31.43 dS·m^−1^
Topsoil Carbonate or lime content (T_CACO_3_)	0.30–31.75%	1.20–14.71%
Precipitation of Driest Month (Bio14)	0.15–2.35 mm	0.15–1.91 mm

**Table 4:** Suitable area of *N. tangutorum* in different periods (unit: ×10^4^ km^2^).

Period	Climate Scenarios	Low Suitable Area	Medium Suitable Area	High Suitable Area	Total Suitable Area	Compare with the Current Increase
Current		108.35	57.73	24.21	190.30	0
2050s	ssp126	105.11	53.91	26.34	185.35	−2.59%
	ssp245	104.58	55.13	26.56	186.27	−2.12%
	ssp370	103.88	53.87	27.07	184.82	−2.88%
	ssp585	106.47	54.82	27.39	188.68	−0.85%
2090s	ssp126	101.77	54.30	22.76	178.83	−6.02%
	ssp245	101.69	62.92	24.24	188.85	−0.76%
	ssp370	103.05	64.10	25.38	192.54	1.18%
	ssp585	103.56	65.72	25.77	195.05	2.50%

**Table 5:** Change of *N. tangutorum* suitable area in different periods (unit: ×10^4^ km^2^).

Period		No Change Area	No Change Rate (%)	Range Contraction Area	Range Contraction Rate (%)	Range Expansion Area	Range Expansion Rate (%)
2050s	SSP126	153.84	80.85	23.68	12.44	19.10	10.03
	SSP245	151.13	79.42	26.39	13.87	20.68	10.87
	SSP370	151.50	79.61	26.02	13.67	18.75	9.85
	SSP585	151.58	79.66	25.94	13.63	22.16	11.64
2090s	SSP126	151.12	79.41	26.40	13.87	19.21	10.09
	SSP245	152.08	79.92	25.44	13.37	23.56	12.38
	SSP370	151.84	79.79	25.68	13.5	27.37	14.38
	SSP585	151.23	79.47	26.30	13.82	28.31	14.88

## Data Availability

The data are contained within the article.
